# Supplementary material for: Reprogramming the antigen specificity of B cells using genome-editing technologies
Source: eLife. 2019 Jan 17;8:e42995. doi: 10.7554/eLife.42995 (PMC6355199; doi:10.7554/eLife.42995)
Supplement: Supplementary file 1. [file elife-42995-supp1.docx]

| **I. gDNA sequence analysis primers** | |
| --- | --- |
| PCR Amplification |  |
| V781-1a | 5’-AGCCCTAAAAAGCATGGGCT-3’ |
| V781-1b | 5’-CTTCTGCACCAAGAGGAGGG-3’ |
| V781-2a | 5’-GCCCTAAAAAGCATGGGCTG-3’ |
| V781-2b | 5’-TCCCCTCCCTTCTGAGTCTG-3’ |
| V781-3a | 5’-GCCATTGTGAGTGAGCCCTA-3’ |
| V781-3b | 5’-AGTCTGCAGTAAACCCCTGC-3’ |
| V434-1a | 5’-ATGTGATTGGCTCCAGGCAT-3’ |
| V434-1a | 5’-CTTCTGCACCAAGAGGAGGG-3’ |
| V434-1a | 5’-GAATGTGATTGGCTCCAGGC-3’ |
| V434-1a | 5’-AGTCTGCAGTAAACCCCTGC-3’ |
| V434-1a | 5’-GCCAGAATGTGATTGGCTCC-3’ |
| V434-1a | 5’-CCAGTGGGGCTTGGTATGTT-3’ |
| Sequencing |  |
| V434 Contig 1, WT Contig 1 | 5’-GCAAATTCCATGTTGCAGTGAGAAGG-3’ |
| V434 Contig 2, WT Contig 2 | 5’-CCTAGTTATGTTGAGTTCCATCAACACTCC-3’ |
| V434 Contig 3, WT Contig 3 | 5’-GCTCTTTGGTTTTCTTTCCACG-3’ |
| V434 Contig 4, WT Contig 4 | 5’-GCACACCCTAGGGTATGTTCTTGC-3’ |
| V434 Contig 5 | 5’-GCAAATCCTCACTTAGGCACCC-3’ |
| V434 Contig 6, WT Contig 7 | 5’-GGGCAAAACCACCTCTTCACAACC-3’ |
| V781 Contig 1 | 5’-AGTCTGCAGTAAACCCCTGC-3’ |
| V781 Contig 2 | 5’-CGTAAACACCAAAACAACACACCC-3’ |
| V781 Contig 3 | 5’-GCATACTACAGAAGTGAGAAACAAAGACAG-3’ |
| V781 Contig 4 | 5’-GCCGTTTCCTGGAGCTCGAGAGGC-3’ |
| V781 Contig 5 | 5’-GAATAGGCAGACATACACGTAGATCAGC-3’ |
| V781 Contig 6 | 5’-CGTAATCCTGTTAGAGGCCACGC-3’ |
| V781 Contig 7 | 5’-GGAGATAAAGCGTATCCTTGG-3’ |
| V781 Contig 8, WT Contig 8 | 5’-CAGATGATGAATTAGAGTCAAGATGGCTGC-3’ |
| V781 Contig 9, V434 Contig 7 | 5’-GACGCCGCATCGGTGATTCGG-3’ |
| WT Contig 5 | 5’-GGTACTATTTAAAAATAACCCAC-3’ |
| WT Contig 6 | 5’-CCCCTGGTTTGTTCAGGCATCTCG-3’ |
| WT Contig 9 | 5’-GGTTAACTCGTTTTCTCTTTGTGATTAAGGAG-3’ |
| **II. Sanger Sequencing of mRNA** | |
| Ramos WT antibody variable region | 5’-AAACACCTGTGGTTCTTCCTCCTCC-3’ |
| PG9 antibody variable region | 5’-GCTGGGTTTTCCTCGTTGCTCTTTTAAG-3’ |
| IgM constant region | 5’-GCGTACTTGCCCCCTCTCAGG-3’ |
| IgG constant region | 5’-GCTTGTGATTCACGTTGCAGATGTAGG-3’ |
| **III. Next Generation Sequencing of Ig mRNA: PCR 1** | |
| Human Heavy Chain |  |
| 5' L-VH 1 | 5’-ACAGGTGCCCACTCCCAGGTGCAG-3’ |
| 5' L-VH 3 | 5’-AAGGTGTCCAGTGTGARGTGCAG-3’ |
| 5' L-VH 4/6 | 5’-CCCAGATGGGTCCTGTCCCAGGTGCAG-3’ |
| 5' L-VH 5 | 5’-CAAGGAGTCTGTTCCGAGGTGCAG-3’ |
| 3' CH1 | 5’-GGAAGGTGTGCACGCCGCTGGTC-3’ |
| Human Kappa |  |
| 5' LVK 1/2 | 5’-ATGAGGSTCCCYGCTCAGCTGCTGG-3’ |
| 5' LVK 3 | 5’-CTCTTCCTCCTGCTACTCTGGCTCCCAG-3’ |
| 5' LVK 4 | 5’-ATTTCTCTGTTGCTCTGGATCTCTG-3’ |
| 3' CK 543 | 5’-GTTTCTCGTAGTCTGCTTTGCTCA-3’ |
| Human Lambda |  |
| 5' LVL 1 | 5’-GGTCCTGGGCCCAGTCTGTGCTG-3’ |
| 5' LVL 2 | 5’-GGTCCTGGGCCCAGTCTGCCCTG -3’ |
| 5' LVL 3 | 5’-GCTCTGTGACCTCCTATGAGCTG -3’ |
| 5' LVL 4/5 | 5’-GGTCTCTCTCSCAGCYTGTGCTG -3’ |
| 5' LVL 6 | 5’-GTTCTTGGGCCAATTTTATGCTG -3’ |
| 5' LVL 7 | 5’-GGTCCAATTCYCAGGCTGTGGTG -3’ |
| 5' LVL 8 | 5’-GAGTGGATTCTCAGACTGTGGTG -3’ |
| 3' CL | 5’-CACCAGTGTGGCCTTGTTGGCTTG-3’ |
| **IV. Off-Target gDNA Analysis** | |
| 1 | 5’-AATAGGCAGACATACACGTAGATCA-3’ |
| 2 | 5’-CAATTATAGGAGCTGAGGGTTCGAG-3’ |
| 3 | 5’-CTCTAGGGCCTTTGTTTTCTGCTA-3’ |
| 4 | 5’-CAGACAATGGTCACTCAAAAGACTC-3’ |
| 5 | 5’-CAGGGTTTTCCCAGTCACGAC-3’ |
| 6 | 5’-TCACACAGGAAACAGCTATGACC-3’ |
